# Geographic and Temporal Differences in Sickle Cell Disease Hospitalizations in New York State

**DOI:** 10.1001/jamanetworkopen.2026.10045

**Published:** 2026-05-01

**Authors:** Chukwuemeka Iloegbu, Jonathan Odumegwu, Joyce Gyamfi, John Patena, Dorice Vieira, Xinyu Wang, Etornam Amesimeku, Prince Amegbor, Andrew Campbell, Folasade Ogunlesi, Uju Ozoh, Emmanuel Peprah

**Affiliations:** 1 Department of Global and Environmental Health, Implementing Sustainable Evidence-Based Interventions Through Engagement Lab, New York University School of Global Public Health, New York, New York; 2 Department of Statistical Science, Baylor University, Waco, Texas; 3 Center for Cancer and Blood Disorders, Children’s National Hospital, Washington, DC; 4 Department of Pediatrics, George Washington University School of Medicine & Health Sciences, Washington, DC; 5 Department of Medicine, College of Medicine, University of Lagos, Lagos, Nigeria

## Abstract

**Importance:**

Sickle cell disease (SCD) disproportionately affects racial and ethnic minority groups in the US and is associated with high levels of morbidity and health care utilization. However, population-level geographic differences and temporal variation in SCD hospitalization outcomes remain incompletely characterized.

**Objective:**

To assess temporal and regional patterns of SCD hospitalizations in New York State from 2009 through 2022.

**Design, Setting, and Participants:**

This retrospective cross-sectional study analyzed inpatient SCD hospitalizations recorded in the New York State Statewide Planning and Research Cooperative System deidentified database between January 1, 2009, and December 31, 2022. The analytic sample included 42 271 hospitalizations after exclusion of records with missing demographic, cost, or facility information. Data analysis was conducted from July 17, 2024, to February 14, 2025.

**Exposures:**

Hospitalization with SCD across 8 state-defined health service areas.

**Main Outcomes and Measures:**

Outcomes included regional distribution of hospitalizations, mean length of stay, mean total charges, and trends in severity of illness and risk of mortality as defined by the All Patient Refined Diagnosis Related Groups classification system. Demographic and regional distributions were compared across years and regions.

**Results:**

Among 42 271 SCD hospitalizations (21 777 female [51.5%]), most occurred among individuals identified as Black (35 318 [83.6%) compared with White (750 [1.8%]), multiracial (242 [0.6%]), and other race or ethnicity 5956 (14.1%) and were aged 18 to 29 (16 794 [39.7%]) or 30 to 49 years (13 480 [31.8%]). New York City accounted for the largest proportion of hospitalizations statewide. There were significant differences in the length of stay and total charges across service areas; Central New York had the longest mean (SD) length of stay of 6.3 (7.3) days, followed by the Hudson Valley (6.2 [7.2]) days, while Long Island had the highest mean (SD) total charges at $59 476.3 ($63 823.5). The proportion of hospitalizations classified as major severity increased from 751 of 5897 (12.7%) in 2009 to 1011 of 3709 (27.3%) in 2022, and the proportion classified as major risk of mortality increased from 170 of 5897 (2.9%) to 469 of 3709 (12.6%) during the same period. Long Island had the highest proportion of hospitalizations with major risk of mortality (93 of 970 [9.6%]), whereas New York City exhibited one of the lower proportions of major risk of mortality (1531 of 27 923 [5.5%]) despite high hospitalization volume.

**Conclusions and Relevance:**

In this cross-sectional study, geographic and temporal differences in SCD hospitalization outcomes were observed across New York State during a 14-year period. These findings suggest the need for region-specific strategies to improve access to specialized care, reduce severe outcomes, and optimize health care resource use for individuals living with SCD.

## Key Points

**Question:**

What were the geographic and temporal patterns of sickle cell disease (SCD) hospitalizations in New York State from 2009 to 2022?

**Findings:**

In this cross-sectional study of 42 271 SCD hospitalizations in New York State, New York City accounted for the largest proportion of admissions, Central New York and the Hudson Valley had the longest mean hospital stays, and Long Island had the highest mean total charges. The proportions of hospitalizations classified as major severity and major risk of mortality increased over time, with the highest levels observed in 2022.

**Meaning:**

These findings suggest the need for region-specific strategies to improve access to specialized care and reduce severe outcomes.

## Teaser

This cross-sectional study assesses trends in outcomes and health care resource utilization among patients with sickle cell disease across different health service areas of New York State.

## Introduction

Sickle cell disease (SCD) is the most common inherited blood disorder in the US, affecting an estimated 100 000 individuals. While it is most prevalent among people of African descent, SCD also affects individuals of Hispanic, Middle Eastern, South Asian, and Southern European backgrounds.^1,2,3^ Individuals with SCD commonly experience chronic hemolytic anemia, recurrent vaso-occlusive episodes, and progressive end-organ damage.^4^ Approximately 10% of all individuals living with SCD in the US reside in New York State, most of whom reside in New York City.^5^ In 2023 alone, approximately 100 infants were born with SCD in New York City, with the highest numbers recorded in the Bronx (18 cases) and Brooklyn (13 cases), while 2602 infants were born with sickle cell trait (960 in the Bronx, 764 in Brooklyn, 317 in Manhattan, 481 in Queens, and 80 in Staten Island).^5^ SCD in New York State is characterized by a disproportionately high prevalence among Black and Hispanic populations, uneven geographic distribution with concentration in specific regions, and a significant burden on the health care system, as evidenced by elevated hospitalization rates and associated health care costs.^5^ Despite effective treatments, individuals with SCD still face frequent hospitalizations, long hospital stays, and high costs.^6,7^

Prior research has documented persistent racial and socioeconomic disparities in the care of individuals with SCD, often resulting in suboptimal disease management and poorer clinical outcomes.^8^ In addition, hospitalizations have been shown to disproportionately affect patients in the lowest income group compared with those in the highest income group.^9^ The management of SCD is further complicated by disparities in access to specialized care, inconsistent uptake of medication treatment that can reduce pain crises such as hydroxyurea, and health care utilization patterns that often rely on home-based opioid management. These factors contribute to delayed presentation and inconsistent use of emergency and inpatient services.^10,11^ Understanding regional differences in length of stay, hospital resource utilization, severity of illness, and mortality among individuals with SCD is critical for identifying gaps in care. These include underutilization of disease-modifying therapies, limited access to adult SCD specialists, and fragmented transition services. Such insights can inform policies aimed at expanding comprehensive care models and shape intervention strategies such as strengthening transition programs, improving access to effective therapies, and enhancing health care professional education to reduce inappropriate reliance on acute care services.^12^

To better understand regional disparities in SCD hospitalizations across New York State, we utilized the publicly available Statewide Planning and Research Cooperative System (SPARCS), a comprehensive, all-payer administrative database that captures inpatient discharge records from health care facilities statewide.^13^ This robust dataset offers a unique opportunity to examine trends in SCD hospitalizations, clinical outcomes, and health care resource utilization during a 14-year period. To our knowledge, this is the first study to systematically analyze the SPARCS dataset to evaluate hospitalization trends among individuals with SCD in New York State. The primary objective of this study was to assess trends in patient outcomes and health care resource utilization among individuals with SCD across different health service areas (HSAs) from January 1, 2009, to December 31, 2022. Specifically, we assessed variations in length of stay (LOS), total charges, total costs, severity of illness, and risk of mortality.

## Methods

### Study Design and Setting

We conducted a retrospective cross-sectional study using inpatient discharge records of patients with SCD from 2009 to 2022, from the New York State SPARCS deidentified dataset. Outcomes included clinical severity and risk of mortality. Since SPARCS is publicly accessible, it excludes unique identifiers, which means hospitalizations cannot be tracked or linked across different encounters for the same individual. As a result, each hospitalization was treated as an analytic unit and repeat admissions by the same individual could not be identified.

The New York University Institutional Review Board determined that this study was exempt from ethics review and the need for informed consent owing to the use of deidentified, publicly available data. The study adhered to the Strengthening the Reporting of Observational Studies in Epidemiology (STROBE) reporting guideline.

### Analytic Sample

**Figure 1.**
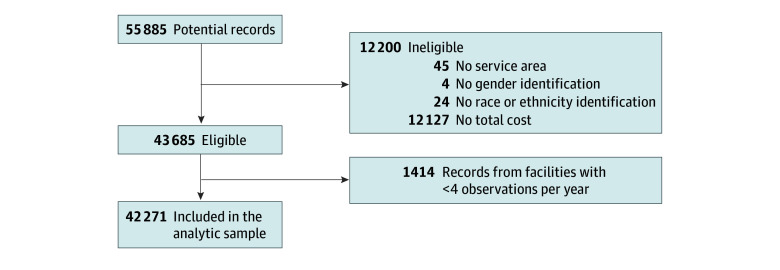
Study Flowchart Data were obtained from the New York State Statewide Planning and Research Cooperative System deidentified database Project-Health Care by Numbers Curriculum.

We identified 55 885 hospitalizations using codes from the *International Classification of Diseases, Ninth Revision*, and *International Statistical Classification of Disease, Tenth Revision*. Inclusion criteria were hospitalizations with a primary or secondary diagnosis of SCD. We excluded 12 200 records because they lacked data on service area (45 [0.08%]), sex (4 [0.007%]), race and ethnicity (24 [0.04%]), or total cost (12 127 [21.7%]). Total cost data were not available for 2018, 2019, and 2021 and accounted for 12 114 missing cases for the total cost. We also excluded 1414 records from hospital facilities with fewer than 4 annual SCD cases, due to limited generalizability. The final analytical sample included 42 271 hospitalizations (Figure 1).

### Variables

#### Severity of Illness and Risk of Mortality

Severity of illness and risk of mortality were measured using the All Patient Refined Diagnosis Related Groups (APR-DRG), version 20.0 (3M Health Information Systems).^14^ Severity of illness reflected physiological decompensation and complexity (eg, loss of organ function); risk of mortality measured the likelihood of in-hospital death. Both are reported on 4 levels: extreme, major, moderate, and minor.^15^ Extreme conditions involve extensive organ dysfunction and life-threatening complications that often necessitate critical care. Major conditions indicate severe symptoms or consequential organ dysfunction requiring intensive medical interventions. Moderate conditions indicate notable but manageable clinical symptoms or risk factors. Minor conditions represent minimal physiologic compromise, limited clinical complexity, and low risk of adverse in-hospital outcomes. Because patients with SCD classified as having an extreme or major condition often shared comparable multisystem complications and resource needs,^16,17^ we combined these conditions into a single category defined as major condition and recategorized severity of illness and risk of mortality into 3 levels (major, moderate, and minor) for analysis.

#### Health Care Utilization

Hospital LOS, total charges, and total costs were derived from administrative data.^18,19^ LOS measured admission to discharge duration. Clinical complexity was characterized using APR-DRG–derived severity of illness and risk of mortality measures, which were used for stratified analyses rather than direct adjustments of LOS. Total charges reflected billed services, which may include room and board, nursing care, medications, or laboratory testing. Estimated hospital costs were derived by applying hospital-specific cost-to-charge ratio to reported total charges. Total cost also approximated the resources required to deliver care such as staff salaries, supplies, or overhead.^20,21,22^

#### Sociodemographic Variables

Sociodemographic variables included age (0-17, 18-29, 30-49, and ≥50 years), race and ethnicity, and sex as reported in the New York’s SPARCS administrative discharge data. Race and ethnicity were classified using categories recorded by health care facilities and reported to SPARCS, including Black, White, multiracial, and not specified. Race and ethnicity were assessed to examine potential differences in hospitalization patterns and outcomes across populations disproportionately affected by SCD. Sex was reported as male or female in SPARCS and reflects administrative classification rather than self-identified gender; 4 records with unidentified sex were treated as missing and were excluded from analysis.

#### Geographic Region

We used New York State Department HSAs as defined by SPARCS to access geographic variations.^23^ Each HSA encompasses multiple counties and licensed health care facilities, with a unique permanent facility identifier enabling analysis of regional disparities in SCD care.

### Missing Data

Hospitalizations with missing demographic, cost, or service area information were excluded from the analytic sample, as described in the sample selection process. Hospitalizations from 2018, 2019, and 2021 were excluded from analyses involving cost and severity-by-year comparisons. Because the SPARCS dataset does not include patient identifiers, loss to follow-up was not applicable to this hospitalization-level analysis.

### Statistical Analyses

Data were analyzed from July 17, 2024, to February 14, 2025. Descriptive statistics were calculated using means and SDs for continuous variables and frequencies and percentages for categorical variables. Because LOS and cost variables were not normally distributed, nonparametric^24^ Kruskal-Wallis tests, followed by the Dunn test for multiple pairwise comparisons, were used to assess differences across HSAs and years of service.^25^ Pearson χ^2^ tests were used to evaluate differences in categorical distributions. Pairwise comparisons of severity and mortality risk proportions were conducted using the Marascuillo procedure.^26,27^ Statistical significance was assessed at a 2-sided *P* < .05. All analyses were conducted using R, version 4.4.0 (R Project for Statistical Computing).^28^

## Results

### Sample Characteristics

After applying exclusion criteria for missing or incomplete demographic, cost, or facility-level data, the final analytic sample included 42 271 hospitalization records. Figure 1 illustrates the study sample selection process using data from SPARCS to analyze SCD hospitalizations in New York State from 2009 to 2022. Facilities with insufficient SCD volume were excluded to reduce instability in regional estimates and to support meaningful comparison across health service areas.^16^ Although this approach may exclude some individual hospitalizations, it prioritizes data from hospitals with consistent volume of SCD-related care during the 14-year study period. This structured approach to sample selection ensured data integrity while optimizing the representativeness of the findings.

### Key Demographic Characteristics

As shown in Table 1, 35 318 hospitalizations (83.6%) involved Black patients, 750 (1.8%) involved White patients, 242 (0.6%) involved multiracial patients, and 5961 (14.1%) involved patients of other race or ethnicity. Most patients were aged 18 to 29 years (16 794 [39.7%]) or 30 to 49 years (13 480 [31.8%]). Geographically, the largest proportion of SCD hospitalizations occurred in New York City (27 923 [66.1%]), followed by the Hudson Valley (4812 [11.4%]). Sex distribution was similar across the sample, with a slightly higher proportion of females (21 777 [51.5%]) than males (20 494 [48.5%). A greater proportion of hospitalizations were classified as major severity of illness (8234 [19.5%]) compared with major risk of mortality (2330 [5.5%])

**Table 1.** Characteristics of the Participants of the SPARCS Data, Stratified by Service Area

Characteristic	Service area									*P* value^a^
	All	Capital-Adirondacks	Central New York	Finger Lakes	Hudson Valley	Long Island	New York City	Southern Tier	Western New York	
Overall, No. (%)^b^	42 271 (100)	1583 (3.7)	1742 (4.1)	2675 (6.3)	4812 (11.4)	970 (2.3)	27 923 (66.1)	109 (0.3)	2457 (5.8)	NA
Age group, No. (%)^c^										
0-17 y	9538 (22.6)	455 (28.7)	288 (16.5)	437 (16.3)	847 (17.6)	133 (13.7)	6878 (24.6)	17 (15.6)	483 (19.7)	<.001
18-29 y	16 794 (39.7)	532 (33.6)	658 (37.8)	1266 (47.3)	1864 (38.7)	328 (33.8)	10 905 (39.1)	60 (55.0)	1181 (48.1)	
30-49 y	13 480 (31.8)	496 (31.3)	700 (40.2)	857 (32.0)	1713 (35.6)	430 (44.3)	8607 (30.8)	31 (28.4)	646 (26.3)	
≥50 y	2459 (5.8)	100 (6.3)	96 (5.5)	115 (4.3)	388 (8.1)	79 (8.1)	1533 (5.5)	1 (0.9)	147 (6.0)	
Sex, No. (%)^c^										
Female	21 777 (51.5)	836 (52.8)	645 (37.0)	1313 (49.1)	2473 (51.4)	526 (54.2)	14 655 (52.5)	63 (57.8)	1266 (51.5)	<.001
Male	20 494 (48.5)	747 (47.2)	1097 (63.0)	1362 (50.9)	2339 (48.6)	444 (45.8)	13 268 (47.5)	46 (42.2)	1191 (48.5)	
Race and ethnicity, No. (%)^c^										
Black	35 318 (83.6)	1431 (90.4)	1565 (89.8)	2540 (95.0)	4084 (84.9)	832 (85.8)	22 450 (80.4)	102 (93.6)	2314 (94.2)	<.001
White	750 (1.8)	102 (6.4)	40 (2.3)	20 (0.7)	109 (2.3)	42 (4.3)	412 (1.5)	3 (2.8)	22 (0.9)	
Multiracial	242 (0.6)	0	1 (0.1)	27 (1.0)	21 (0.4)	6 (0.6)	179 (0.6)	0	8 (0.3)	
Other	5961 (14.1)	50 (3.2)	136 (7.8)	88 (3.3)	598 (12.4)	90 (9.3)	4882 (17.5)	4 (3.7)	113 (4.6)	
Hospital LOS, mean (SD), d	5.6 (6.4)	5.0 (4.5)	6.3 (7.3)	5.6 (7.0)	6.2 (7.2)	5.5 (5.2)	5.5 (6.2)	4.2 (3.7)	5.3 (6.2)	<.001
Total charges, mean (SD), $	38 644.8 (77 921.1)	22 744.3 (22 140.6)	29 885.3 (36 235.3)	14 493.7 (18 742.5)	46 871.6 (94 266.9)	59 476.3 (63 823.5)	42 157.9 (84 390.8)	13 309.6 (10 756.5)	18 255.5 (33 807.4)	<.001
Total cost, mean (SD), $	13 214.7 (22 743.6)	7589.2 (7455.7)	11 959.8 (14 349.4)	7235.5 (8133.0)	13 475.9 (23 423.6)	14 017.2 (14 504.0)	14 474.0 (25 057.5)	6445.0 (5465.7)	9398.8 (16 003.1)	<.001
Severity of illness, No. (%)^c^^,^^d^										
Minor	17 130 (40.5)	655 (41.4)	736 (41.7)	1313 (49.1)	2044 (42.5)	356 (36.7)	11 172 (40.0)	39 (35.8)	815 (33.2)	<.001
Moderate	16 907 (40.0)	669 (42.3)	757 (43.5)	1019 (38.1)	1961 (40.8)	408 (42.1)	10 945 (39.2)	46 (42.2)	1102 (44.9)	
Major	8234 (19.5)	259 (16.4)	249 (14.3)	343 (12.8)	807 (16.8)	206 (21.2)	5806 (20.8)	24 (22.0)	540 (22.0)	
Risk of mortality, No. (%)^c^^,^^d^										
Minor	34 695 (82.1)	1336 (84.4)	1502 (86.2)	2224 (83.1)	3966 (82.4)	703 (72.5)	22 857 (81.9)	82 (75.2)	2025 (82.4)	<.001
Moderate	5246 (12.4)	170 (10.7)	157 (9.0)	315 (11.8)	579 (12.0)	174 (17.9)	3535 (12.7)	20 (18.3)	296 (12.0)	
Major	2330 (5.5)	77 (4.9)	83 (5.8)	136 (5.1)	267 (5.5)	93 (9.6)	1531 (5.5)	7 (6.6)	136 (5.5)	

Abbreviations: LOS, length of stay; NA, not applicable.

^a^ Calculated using Kruskal-Wallis rank sum test or Pearson χ^2^ test.

^b^ Percentages are calculated by the row.

^c^ Percentages are calculated by the column.

^d^ Categories are based on All Patient Refined Diagnosis Related Groups classifications assigned at hospital discharge.

The overall mean (SD) LOS was 5.6 (6.4) days. Central New York had the longest mean (SD) LOS of 6.3 (7.3) days, followed by the Hudson Valley (6.2 [7.2] days). In contrast, the Southern Tier had the shortest mean (SD) LOS of 4.2 (3.7) days. As shown in Table 2, the mean (SD) LOS remained relatively stable during the 14-year period, increasing modestly from 5.2 (5.2) days in 2017 to 6.1 (7.9) days in 2022.

**Table 2.** Characteristics of the Participants of the Statewide Planning and Research Cooperative System Data, Stratified by Year of Service

Characteristic	Service year^a^												*P* value^b^
	All (n = 42 271)	2009 (n = 5897)	2010 (n = 5995)	2011 (n = 2008)	2012 (n = 2601)	2013 (n = 2976)	2014 (n = 3063)	2015 (n = 2975)	2016 (n = 2850)	2017 (n = 4300)	2020 (n = 5897)	2022 (n = 3709)	
Age group, No. (%)													
0-17 y	9538 (22.6)	1489 (25.3)	1428 (23.8)	450 (22.4)	643 (24.7)	727 (24.4)	766 (25.0)	797 (26.8)	737 (25.9)	926 (21.5)	837 (14.2)	738 (19.9)	<.001
18-29 y	16 794 (39.7)	2366 (40.1)	2374 (39.6)	809 (40.3)	1066 (41.0)	1215 (40.8)	1269 (41.4)	1227 (41.2)	1212 (42.5)	1713 (39.8)	2282 (38.7)	1261 (34.0)	
30-49 y	13 480 (31.9)	1831 (31.0)	1922 (32.1)	621 (30.9)	751 (28.9)	850 (28.6)	864 (28.2)	800 (26.9)	763 (26.8)	1426 (33.2)	2302 (39.1)	1350 (36.4)	
≥50 y	2459 (5.8)	211 (3.6)	271 (4.5)	128 (6.4)	141 (5.4)	184 (6.2)	164 (5.4)	151 (5.1)	138 (4.8)	235 (5.5)	476 (8.1)	360 (9.7)	
Sex, No. (%)													
Female	21 777 (51.5)	3136 (53.2)	3312 (55.2)	968 (48.2)	1304 (50.1)	1472 (49.5)	1557 (50.8)	1501 (50.5)	1382 (48.5)	2228 (51.8)	2997 (50.8)	1920 (51.8)	<.001
Malen	20 494 (48.5)	2761 (46.8)	2683 (44.8)	1040 (51.8)	1297 (49.9)	1504 (50.5)	1506 (49.2)	1474 (49.5)	1468 (51.5)	2072 (48.2)	2900 (49.2)	1789 (48.2)	
Race and ethnicity, No. (%)													
Black	35 318 (83.6)	4894 (83.0)	5028 (83.9)	1787 (89.0)	2269 (87.2)	2455 (82.5)	2587 (84.5)	2433 (81.8)	2350 (82.5)	3307 (76.9)	5072 (86.0)	3136 (84.6)	<.001
White	750 (1.8)	158 (2.7)	136 (2.3)	39 (1.9)	53 (2.0)	35 (1.2)	23 (1.1)	42 (1.4)	57 (2.0)	89 (2.1)	88 (1.5)	30 (0.8)	
Multiracial	242 (0.6)	0	0	0	0	0	7 (0.2)	21 (0.7)	19 (0.7)	103 (2.4)	62 (1.1)	30 (0.8)	
Other	5961 (14.1)	845 (14.3	831 (13.9)	182 (9.1)	279 (10.7)	486 (16.3)	446 (14.6)	479 (16.1)	424 (14.9)	801 (18.6)	675 (11.4)	513 (13.8)	
Hospital LOS, mean (SD), d	5.6 (6.4)	5.5 (6.0)	5.5 (5.3)	5.7 (6.6)	5.5 (6.0)	5.5 (6.5)	5.6 (6.6)	5.7 (7.8)	5.2 (6.5)	5.2 (5.2)	5.7 (6.4)	6.1 (7.9)	<.001
Total charges, mean (SD), $	38 644.8 (77 921.1)	26 424.5 (39 110.9)	27 078.1 (34 678.6)	25 287.1 (35 297.3)	30 667.9 (43 165.0)	34 300.5 (70 598.2)	35 912.3 (77 668.3)	40 918.7 (137 301.1)	38 492.3 (95 085.6)	41 716.6 (84 848.1)	52 484.7 (70 630.0)	68 064.8 (117 177.9)	<.001
Total cost, mean (SD), $	13 214.7 (22 743.6)	11 008.9 (17 857.0)	11 209.0 (14 296.4)	11 863.6 (19 262.2)	9703.0 (12 290.5)	10 812.9 (21 215.7)	11 541.0 (19 186.7)	13 307.9 (37 186.5)	12 667.2 (26 718.1)	13 666.1 (21 223.2)	16 543.3 (21 532.8)	20 997.3 (32 571.1)	<.001
Severity of illness, No. (%)													
Minor	17 130 (40.5)	2881 (48.9)	2725 (45.5)	754 (37.5)	949 (36.5)	1294 (43.5)	1234 (40.3)	1256 (42.2)	1173 (41.2)	1679 (39.0)	1971 (33.4)	1214 (32.7)	<.001
Moderate	16 907 (40.0)	2265 (38.4)	2349 (39.2)	847 (42.1)	1080 (41.5)	1242 (41.7)	1270 (41.5)	1126 (37.8)	1091 (38.3)	1626 (37.8)	2527 (42.9)	1484 (40.0)	
Major	8234 (19.5)	751 (12.7)	921 (15.4)	407 (20.3)	572 (22.0)	440 (14.8)	559 (18.3)	593 (19.9)	586 (20.6)	995 (23.1)	1399 (23.7)	1011 (27.3)	
Risk of mortality, No. (%)^c^													
Minor	34 695 (82.1)	5237 (88.8)	5242 (87.4)	1664 (82.9)	2154 (82.8)	2549 (85.7)	2574 (84.0)	2464 (82.8)	2449 (85.9)	3583 (83.3)	4278 (72.5)	2501 (67.4)	<.001
Moderate	5246 (12.4)	490 (8.3)	533 (8.9)	245 (12.2)	329 (12.6)	335 (11.3)	357 (11.7)	369 (12.4)	271 (9.5)	504 (11.7)	1074 (18.2)	739 (19.9)	
Major	2330 (5.5)	170 (2.9)	220 (3.7)	99 (4.9)	118 (4.5)	92 (3.1)	132 (4.3)	142 (4.8)	130 (4.6)	213 (5.0)	545 (9.2)	469 (12.6)	
Service area, No. (%)^c^													
Capital-Adirondacks region	1583 (3.7)	4 (0.1)	4 (0.1)	230 (11.5)	244 (9.4)	185 (6.2)	191 (6.2)	197 (6.6)	172 (6.0)	85 (2.0)	176 (3.0)	95 (2.6)	<.001
Central New York	1742 (4.1)	0	1 (0.02)	255 (12.7)	263 (10.1)	279 (9.4)	278 (9.1)	211 (7.1)	184 (6.5)	76 (1.8)	127 (2.2)	68 (1.8)	
Finger Lakes	2675 (6.3)	4 (0.1)	1 (0.02)	299 (14.9)	280 (10.8)	337 (11.3)	355 (11.6)	393 (13.2)	350 (12.3)	190 (4.4)	281 (4.8)	185 (5.0)	
Hudson Valley	4812 (11.4)	352 (6.0)	395 (6.6)	518 (25.8)	550 (21.1)	529 (17.8)	515 (16.8)	535 (18.0)	566 (19.9)	285 (6.6)	361 (6.1)	206 (5.6)	
Long Island	970 (2.3)	33 (0.6)	45 (0.8)	0	0	0	0	0	0	251 (5.8)	382 (6.5)	259 (7.0)	
New York City	27 923 (66.1)	5502 (93.3)	5548 (92.5)	408 (20.3)	990 (38.1)	1322 (44.4)	1385 (45.2)	1322 (44.4)	1210 (42.5)	3256 (75.7)	4238 (71.9)	2742 (73.9)	
Southern Tier	109 (0.3)	2 (0.03)	1 (0.02)	11 (0.5)	10 (0.4)	16 (0.5)	11 (0.4)	14 (0.5)	14 (0.5)	0	22 (0.4)	8 (0.2)	
Western New York	2457 (5.8)	0	0	287 (14.3)	264 (10.1)	308 (10.3)	328 (10.7)	303 (10.2)	354 (12.4)	157 (3.7)	310 (5.3)	146 (3.9)	

Abbreviation: LOS, length of stay.

^a^ Years 2018, 2019, and 2021 were dropped due to missingness of total costs in those years of service in the Statewide Planning and Research Cooperative System dataset.

^b^ Calculated using Kruskal-Wallis rank sum test or Pearson χ^2^ test.

^c^ Categories are based on All Patient Refined Diagnosis Related Groups classifications assigned at hospital discharge.

The mean (SD) overall hospital charges were $38 644.8 ($77 921.1) and overall total costs were $13 214.7 ($22 743.6). Across the HSAs, Long Island had the highest mean (SD) total charges ($59 476.3 [$63 823.5]), whereas New York City had the highest mean (SD) total costs ($14 474.0 [$25 057.5]). Table 2 shows that the mean total charges and costs remained relatively stable during the years of service, with overall mean (SD) values of $38 644.8 ($77 921.1) and $13 214.7 ($22 743.6), respectively.

### Patient Outcomes: SOI by Service Area and Year

**Figure 2.**
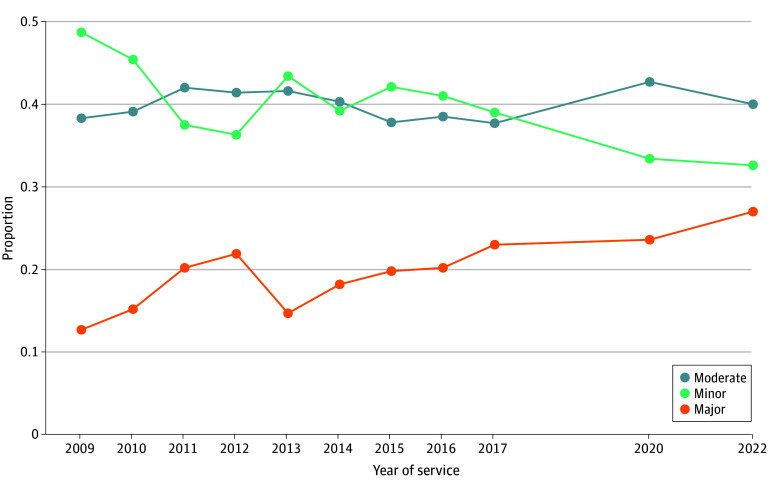
Line Graph of Trends in Severity of Sickle Cell Disease Hospitalizations in New York State, 2009 to 2022 Data were obtained from the All Patient Refined Diagnosis Related Groups system. Numbers and percentages are found in Table 2.

Tables 1 and 2, eFigure 1 in Supplement 1, and Figure 2 present trends during the 14-year study period in socioeconomic and demographic characteristics, severity of illness, and mortality risk among SCD hospitalizations across New York State health service areas. The Southern Tier and Western New York had the highest proportions of hospitalizations classified as major severity (24 of 109 [22.0%] and 540 of 2457 [22.0%], respectively), followed by Long Island (206 of 970 [21.2%]) and New York City (5806 of 27 923 [20.8%]). Over time, the proportion of hospitalizations classified as major severity increased from 751 of 5897 (12.7%) in 2009 and 559 of 3063 (18.3%) in 2014 to its highest recorded level at 1011 of 3709 (27.3%) in 2022 (Figure 2).

**Figure 3.**
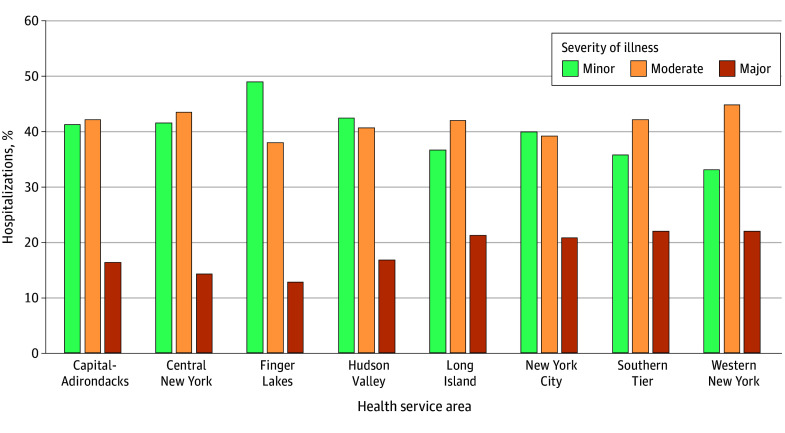
Geographic Map of Severity Levels of Hospitalizations by Service Area Numbers and percentages are provided in Table 1. Statistical tests confirm significant differences across regions for each severity category, reflecting true regional disparities in disease manifestation. These patterns may indicate differences in access to specialized care, socioeconomic conditions, or health care delivery systems.

Statistically significant differences were observed in the distribution of severity classification across health service areas, with the following test statistics for the severity categories: major (χ^2^_7_ = 179.00), moderate (χ^2^_7_ = 51.08), and minor (χ^2^_7_ = 153.96) (*P* < .001 for all). Differences in severity distribution across years were also statistically significant with the following text statistics: major (χ^2^_10_ = 540.42), moderate (χ^2^_10_ = 58.10), and minor (χ^2^_10_ = 494.0) (*P* < .001 for all). Figure 3 shows the proportion of SCD hospitalizations by severity classification, summarizing the distributions reported in eTable 1 in Supplement 1 and highlighting regional variation in severity profiles.

Pairwise comparisons using Marascuilo procedure identified statistically significant differences in the proportion of hospitalizations classified as major severity between several HSAs. Notable differences were observed between the Capital-Adirondacks region and New York City, the Capital-Adirondacks region and Western New York, Central New York and Long Island, and Central New York and New York City (eTable 3 in Supplement 1). Statistically significant differences in the proportion of major severity hospitalizations were also observed across years, including differences between 2009 and 2010, 2011, 2014, and 2022 (Table 2 and eTables 2 and 4 in Supplement 1).

### Patient Outcomes: Risk of Mortality by Service Area and Year

Tables 1 and 2 summarize variation in the distribution of mortality risk classifications among SCD hospitalizations across New York State HSAs and over time. Overall, 2330 hospitalizations (5.5%) were classified as major risk of mortality (Table 1). Long Island had the highest proportion of hospitalizations classified as major mortality risk (93 of 970 [9.6%]), followed by Western New York (136 of 2457 [5.5%]) and the Southern Tier (7 of 109 [6.6%]). Although New York City accounted for the largest number of SCD hospitalizations statewide, it exhibited one of the lower proportions of hospitalizations classified as major mortality risk (1531 of 27 923 [5.5%])compared with other service areas (Table 1).

During the years of service, the proportion of hospitalizations classified as major mortality risk increased from 170 of 5897 (2.9%) in 2009 to a peak of 469 of 3709 (12.6%) in 2022 (Table 2). During the same period, the proportion classified as minor mortality risk declined from 5237 of 5897 (88.8%) in 2009 to 2501 of 3709 (67.4%), while the proportion classified as moderate mortality risk increased from 490 of 5897 (8.3%) to 739 of 3709 (19.9%). These distributions reflect temporal changes in mortality risk classification among hospitalized individuals with SCD during the study period.

### Hospital LOS

Hospital LOS varied significantly (*P* < .001) across health service areas and severity of illness classifications (eTable 1 in Supplement 1). Among hospitalizations classified as major severity of illness, Central New York had the longest mean (SD) LOS (12.5 [14.2] days), followed by the Finger Lakes (11.06 [14.0] days) and the Hudson Valley (11.1 [13.0] days). The Southern Tier had the shortest mean (SD) LOS among hospitalizations classified as major severity (5.5 [3.8] days). For hospitalizations classified as moderate severity cases, Central New York again had the longest mean (SD) LOS (6.2 [5.5] days), followed by the Hudson Valley (6.1 [5.7] days), while the Southern Tier had the shortest LOS (4.1 [3.9] days). Among hospitalizations classified as minor severity cases, the Hudson Valley had the longest mean (SD) LOS (4.3 [3.4] days), whereas the Capital-Adirondacks region had the shortest (3.6 [2.7] days). Across the years of service (eTable 2 in Supplement 1), mean (SD) LOS for hospitalizations classified as major severity varied, with the longest observed in 2009 (10.2 [11.7] days) and the shortest in 2017 (7.8 [8.0] days). eFigure 2 in Supplement 1 illustrates the variation in mean LOS over time by severity classification across health service areas. No statistically significant differences in LOS trends over time were observed for hospitalizations classified as moderate and minor severity.

Dunn pairwise comparisons (eTable 5 in Supplement 1) also identified statistically significant differences in LOS between several HSAs. Notable differences were observed between the Capital-Adirondacks region and Central New York, the Capital-Adirondacks region and the Hudson Valley, Central New York and the Finger Lakes, Central New York and New York City, Central New York and Western New York, the Finger Lakes and the Hudson Valley, the Hudson Valley and New York City, the Hudson Valley and the Southern Tier, and the Hudson Valley and Western New York. Also, there were some differences in LOS between Central New York and the Southern Tier and between the Hudson Valley and Long Island.

## Discussion

To our knowledge, this study is the first to use the SPARCS dataset to conduct a comprehensive evaluation of SCD hospitalizations across New York State from 2009 to 2022. Using data from 42 271 hospitalizations, we observed persistent geographic differences in health care utilization, severity of illness, and mortality risk classifications over time.^29^

Most of the hospitalizations occurred among Black individuals (83.6%), which is consistent with known racial distribution of SCD in the US. In New York State newborn screening data, 86% of infants diagnosed with SCD were born to non-Hispanic Black mothers, indicating a predominance of Black individuals among those living with SCD in this region.^30^ Young adults (aged 18-29 years) accounted for the largest proportion of hospitalizations (39.7%) followed by those aged 30 to 49 years (31.8%). These age patterns are consistent with prior literature describing the transition from pediatric to adult care as a period when individuals with SCD experience increased health care utilization, potentially reflecting gaps in access to specialized services, changes in care coordination, and broader system-level challenges.

Substantial regional variations were observed across New York State. Central New York (mean [SD] LOS, 6.3 [7.3] days) and the Hudson Valley (mean [SD] LOS, 6.2 [7.2] days) had the longest LOS, whereas the Southern Tier had the shortest (4.2 [3.7] days). These differences may reflect variation in clinical complexity, as captured by APR-DRG severity classifications, as well as regional differences in care delivery patterns. Long Island reported the highest mean (SD) total charges ($59 476.3 [$63 823.5]), while New York City had the highest mean (SD) total hospital costs ($14 474.0 [$25 057.5]), indicating regional variation in resource utilization and financial burden.^31^

Differences were also observed in severity of illness and mortality risk classifications. The Southern Tier and Western New York had the highest proportion of hospitalizations classified as major severity (22.0% for both), while Long Island had the highest proportion classified as major mortality risk (9.6%), exceeding the statewide average of 6%. These patterns may reflect multiple underlying factors. Differences in the age distribution, particularly the higher proportion of adults 30 years or older among hospitalized individuals on Long Island as well as regional variation in access to high-quality primary and specialty SCD care, may contribute to delayed disease management and greater severity at the time of hospitalization in some regions. By 2022, the proportions of hospitalizations classified as major severity and major mortality risk increased to 27.3% and 12.6%, respectively. These temporal patterns coincided with the COVID-19 pandemic period, during which disruptions in health care access and continuity for individuals with SCD were widely reported.^32,33^

In the US, more than 98% of pediatric patients with SCD survive into adulthood, reflecting substantial advances in disease management and supportive care.^34,35^ As individuals with SCD age, cumulative organ damage, an established age-dependent risk factor in SCD, contributes to the increasing morbidity and mortality observed in aging patients with SCD beyond the second decade of life.^36^ Consistent with this pattern, New York City, which had the highest proportion of pediatric SCD hospitalizations, had a lower proportion of hospitalizations classified as major mortality risk than Long Island, a region with a higher proportion of adult SCD hospitalizations.

Our findings are consistent with national literature describing higher health care utilization and elevated mortality risk classifications among young adults with SCD, particularly during the transition to adult care. For example, Fasipe et al^37^ examined more than 37 million hospital encounters among individuals aged 16 to 24 years and reported that, although overall in-hospital mortality remained stable, hospitalizations among individuals with SCD increased annually, with those aged 19 to 24 years having higher odds of in-hospital death compared with adolescents aged 16 to 18 years. Prior studies^10,38^ have similarly reported substantial emergency department use and inpatient hospitalizations among individuals with SCD, with higher mortality observed among adults aged 20 to 24 years. Insurance coverage and continuity of care have been identified in prior studies as important contextual factors associated with health care utilization among individuals with SCD. For example, Medicaid-enrolled individuals with SCD have been reported to experience higher mortality rates and increased use of inpatient and emergency services compared with the general population.^29,39,40,41^

Regional patterns observed in this study suggest that Long Island and New York City, which had higher mean charges and costs, may be managing hospitalizations characterized by greater clinically complexity or differences in care delivery practices. These patterns may also reflect regional variation in payer mix, including Medicaid enrollment and access to specialized SCD care. Evidence from the National Health Interview Survey and reports from the Centers for Medicare & Medicaid Services indicate that individuals with SCD frequently face chronic comorbid conditions, insurance instability, limited access to specialist care (ie, hematologist), and financial hardship, factors that have been associated with recurrent health care utilization in prior research.^40,41,42^

The COVID-19 pandemic provides additional context for the temporal patterns observed in this study. Despite advances in disease-modifying therapies for SCD,^43^ multiple studies have documented disruptions to health care access during the pandemic. Prior evidence indicates that individuals with SCD experienced higher rates of severe COVID-19 complications and hospitalization compared with the general population.^44^ Data from the Surveillance Epidemiology of COVID-19 Under Research Exclusion–SCD registry further documented high hospitalization rates among individuals with SCD who contracted COVID-19, including both adults and children.^45^ These observations provide important temporal context for the increases in severity and mortality classifications observed in later study years, although direct comparison with COVID-19 outcomes in the general New York State population was not possible within the constraints of the SPARCS dataset.

Future research should further explore multilevel factors associated with regional variation in SCD outcomes, including hospital infrastructure, clinician experience, and the structural inequities within health care delivery systems. Integrating inpatient, outpatient, and emergency department data could provide a more complete picture of health care utilization patterns and identify critical gaps in care. Qualitative research may also offer important insights into patient experiences and systemic barriers influencing reliance on acute care services.

In this retrospective study of SCD hospitalizations in New York State from 2009 to 2022, substantial geographic and temporal differences in hospitalizations were observed. The findings demonstrate pronounced regional variation in health care utilization, severity classifications, and mortality risk across the state. These patterns underscore the importance of regionally tailored health policy approaches that account for differences in access to specialized care, resource availability, and clinical burden. Strategies aimed at strengthening access to specialized SCD care, improving care coordination, and structural barriers within health systems may support more equitable outcomes. Addressing these disparities will likely require a multilevel approach that integrates improvements in hospital-based care with broader systemic reforms to promote continuous and comprehensive care for individuals with SCD across the lifespan.

### Limitations

This study has several limitations. As with all analyses of administrative data, misclassification or coding errors are possible. The SPARCS dataset does not include patients’ identifiers, limiting the ability to examine longitudinal outcomes such as repeat hospitalizations, interfacility transfers, or disease progression over time. As a result, patterns of care continuity or recurrent admissions could not be directly assessed. Consequently, each hospitalization was analyzed as a separate event, and patterns of frequent utilization among individuals with SCD could not be assessed. This limitation may result in overestimation of hospitalization counts and percentages if a subset of patients contributed multiple admissions during the study period. Additionally, although the study period includes the COVID-19 pandemic, the publicly available SPARCS dataset does not allow for estimation of the relative risk of severe COVID-19 infection or COVID-19–related mortality among individuals with SCD compared with the general New York State population. The dataset lacks population-level denominators, outpatient case capture, and individual-level linkage across hospitalizations, limiting direct comparative risk assessment.

In addition, the publicly available SPARCS inpatient dataset lacks detailed clinical presentation variables, including physiologic measures at hospital arrival (eg, blood pressure or heart rate), limiting assessment of acute clinical acuity beyond administrative severity classifications. Medication and treatment exposure data, including use of disease-modifying therapies such as hydroxyurea, are also unavailable, precluding evaluation of treatment effects on hospitalization severity or outcomes. Furthermore, the lack of granular socioeconomic and residential information restricts the assessment of neighborhood-level factors, environmental exposures, and other structural determinants that may contribute to regional differences in hospitalization patterns and outcomes. Nevertheless, the large sample size and comprehensive statewide coverage of SPARCS provide a robust framework for examining regional and temporal variation in SCD hospitalizations at the population level.

## Conclusions

This cross-sectional study provides a comprehensive assessment of SCD hospitalizations in New York State during a 14-year period and identifies substantial regional variation in health care utilization. New York City accounted for the largest proportion of hospitalizations, while Central New York and the Hudson Valley reported had the longest mean hospital stays. Long Island had the highest mean total charges per hospitalization. Over time, the proportion of hospitalizations classified as major severity and major mortality risk increased, with the highest proportions observed in 2022.

These findings demonstrate that the burden of SCD hospitalizations is unevenly distributed across regions in New York State. Areas characterized by lower access to specialized SCD care exhibit higher proportions of hospitalizations with greater severity classifications, longer lengths of stay, and higher costs. By leveraging statewide administrative data from SPARCS, this analysis provides insights into the temporal and geographic patterns of SCD-related hospitalizations and highlights priorities for future research. Efforts to reduce regional inequities in SCD care may benefit from expanded access to specialized care, enhancing clinician training, and improved availability of disease-modifying and curative therapies. Regionally informed policy approaches and cross-sector collaboration may support more equitable and continuous care for individuals living with SCD across New York State.

## Notes

Supplement 1. **eFigure 1.** Severity of Sickle Cell Disease in New York State (2009-2022) **eFigure 2.** Mean Length of Stay by Severity and Service Area **eTable 1.** Proportions of Severity levels of SCD With Their Length of Hospital Stay Across the Health Service Areas (2009-2022) **eTable 2.** Proportions of Severity levels of SCD With Length of Hospital Stay Across the Year of Service (2009-2022) **eTable 3.** Marascuilo Comparison of Severity Levels of SCD Across the Health Service Areas (2009-2022) **eTable 4.** Marascuilo Comparison of Severity Levels of SCD in New York State Across the Year of Service (2009-2022) **eTable 5.** Dunn HSD Multiple Comparisons for Length of Stay, Total Charges, and Total Costs Across Health Service Areas

Supplement 2.
Data Sharing Statement
