# Supplementary material for: Geographic and Temporal Differences in Sickle Cell Disease Hospitalizations in New York State
Source: JAMA Netw Open. 2026 May 1;9(5):e2610045. doi: 10.1001/jamanetworkopen.2026.10045 (PMC13135208; doi:10.1001/jamanetworkopen.2026.10045)
Supplement: Supplement 1. — eFigure 1. Severity of Sickle Cell Disease in New York State (2009-2022) eFigure 2. Mean Length of Stay by Severity and Service Area eTable 1. Proportions of Severity levels of SCD With Their Length of Hospital Stay Across the Health Service Areas (2009-2022) eTable 2. Proportions of Severity levels of SCD With Length of Hospital Stay Across the Year of Service (2009-2022) eTable 3. Marascuilo Comparison of Severity Levels of SCD Across the Health Service Areas (2009-2022) eTable 4. Marascuilo Comparison of Severity Levels of SCD in New York State Across the Year of Service (2009-2022) eTable 5. Dunn HSD Multiple Comparisons for Length of Stay, Total Charges, and Total Costs Across Health Service Areas [file jamanetwopen-e2610045-s001.pdf]

Supplementary Online Content

Iloegbu C, Odumegwu J, Gyamfi J, et al. Geographic and temporal differences in sickle cell disease hospitalizations. *JAMA Netw Open.* 2026;9(5):e2610045. doi:10.1001/jamanetworkopen.2026.10045

eFigure 1. Severity of Sickle Cell Disease in New York State (2009-2022)

eFigure 2. Mean Length of Stay by Severity and Service Area

eTable 1. Proportions of Severity levels of SCD With Their Length of Hospital Stay Across the Health Service Areas (2009-2022)

eTable 2. Proportions of Severity levels of SCD With Length of Hospital Stay Across the Year of Service (2009-2022)

eTable 3. Marascuilo Comparison of Severity Levels of SCD Across the Health Service Areas (2009-2022)

eTable 4. Marascuilo Comparison of Severity Levels of SCD in New York State Across the Year of Service (2009-2022)

eTable 5. Dunn HSD Multiple Comparisons for Length of Stay, Total Charges, and Total Costs Across Health Service Areas

This supplementary material has been provided by the authors to give readers additional information about their work.

© 2026 Iloegbu C et al. *JAMA Network Open*.

eFigure 1. Severity of Sickle Cell Disease in New York State (2009-2022)

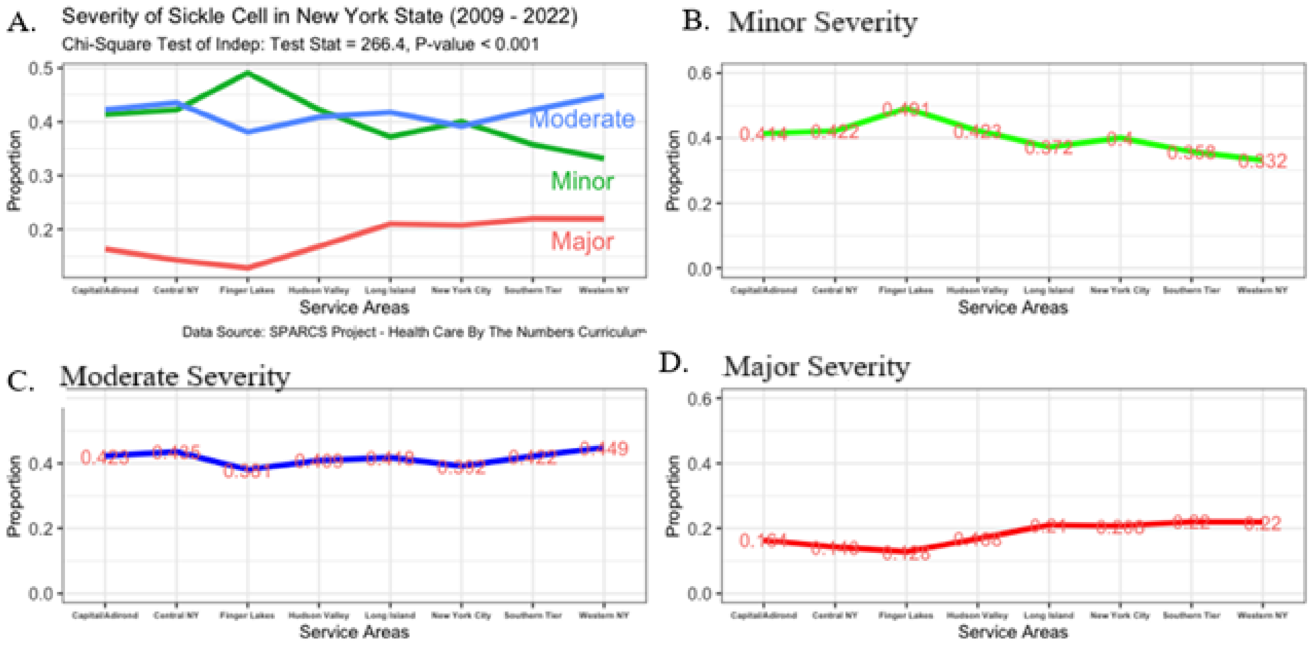

This figure depicts the proportional distribution of severity levels among individuals hospitalized with SCD across New York State service areas between 2009 and 2022. Panel 2A presents an aggregated line graph comparing the three severity categories—Minor (green), Moderate (blue), and Major (red)—across all eight service areas. Panels 2B, 2C, and 2D separately highlight the distribution trends for Minor, Moderate, and Major severity cases, respectively. The data are sourced from the SPARCS Project and were analyzed using a Chi-square test, which yielded a significant result ($\chi^2 = 266.4$, $p < 0.001$), indicating that severity proportions significantly varied by region.

Data from the years 2018, 2019, and 2021 were excluded from this figure due to missing total cost information in the SPARCS dataset, which may influence temporal and spatial continuity in trend interpretations.

© 2026 Iloegbu C et al. *JAMA Network Open*.

eFigure 2. Mean Length of Stay by Severity and Service Area

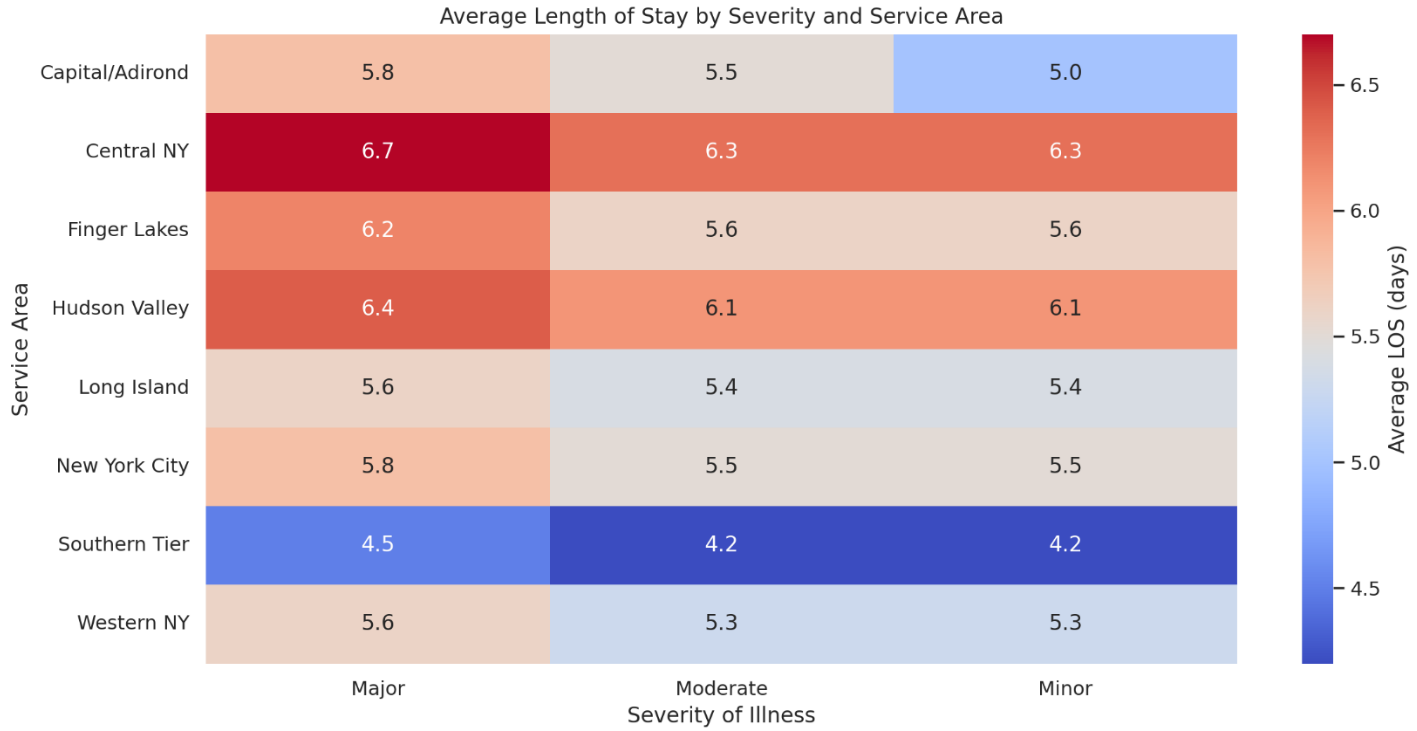

This figure displays a heatmap of the average length of hospital stay (LOS) for sickle cell disease (SCD) hospitalizations by severity level across selected years from 2009 to 2022. The color gradient in the heatmap represents the proportion of SCD hospitalizations classified under each severity level (Major, Moderate, Minor) across New York State service areas. Shades of blue indicate lower proportions, while shades of red reflect higher proportions. This visualization, derived from eTable 2, captures temporal trends in healthcare utilization for patients with SCD, stratified by major, moderate, and minor severity of illness. Notably, patients classified under the major severity category consistently experienced longer hospital stays, with the highest average LOS observed in 2010 (9.35 days) and 2009 (10.24 days). Conversely, individuals with minor severity generally had shorter LOS, averaging between 3.49 and 4.06 days throughout the observation period. Over time, a downward trend in LOS for major severity cases is evident, particularly between 2009 and 2017, followed by a modest increase in later years such as 2022. Meanwhile, LOS for moderate severity cases remained

© 2026 Iloegbu C et al. *JAMA Network Open*.

relatively stable, fluctuating between 5.02 and 5.74 days. The Southern Tier and Western NY service areas had the lowest LOS for all severity levels, suggesting potential differences in care delivery or case management. Chi-square tests for equality of proportions revealed statistically significant differences across years for all severity categories, confirming that temporal shifts in severity distribution and LOS were not due to random variation.

© 2026 Iloegbu C et al. *JAMA Network Open*.

eTable 1. Proportions of Severity levels of SCD With Their Length of Hospital Stay Across the Health Service Areas (2009-2022)

Service Area	Total	Major			Moderate			Minor		
		n	Proportion (%)	Mean (SD) Length of Stay	n	Proportion (%)	Mean (SD) Length of Stay	n	Proportion (%)	Mean (SD) Length of Stay
Capital/Adirond	1,583	259	16.4	8.2 (6.7)	669	42.3	5.3 (4.2)	655	41.4	3.6 (2.7)
Central NewYork	1,742	249	14.3	12.5 (14.2)	757	43.5	6.2 (5.5)	736	42.3	4.2 (3.2)
Finger Lakes	2,675	343	12.8	11.1 (14.0)	1,019	38.1	5.8 (5.6)	1,313	49.1	4.1(4.0)
Hudson Valley	4,812	807	16.8	11.1 (13.0)	1,961	40.8	6.1 (5.7)	2,044	42.5	4.3 (3.4)
Long Island	97,0	206	21.2	8.2 (7.5)	408	42.1	5.6 (4.8)	356	36.7	3.7 (3.0)
New York City	27.923	5,806	20.8	8.9 (10.5)	10,945	39.2	5.3 (4.5)	11,172	40.0	3.8 (3.3)
Southern Tier	109	24	22.0	5.46 (3.8)	46	42.2	4.1 (3.9)	39	35.8	3.6 (3.2)
Western NY	2,457	540	22.0	8.5 (10.4)	1,102	44.9	5.0 (4.45)	815	33.1	3.6 (2.9)
**Test for equality of proportions**		$\chi^2_{(7)}$ = **179.0, p-value < 0.001**			$\chi^2_{(7)}$ = **51.1, p-value < 0.001**			$\chi^2_{(7)}$ = **154.0, p-value < 0.001**		

© 2026 Iloegbu C et al. *JAMA Network Open*.

eTable 2. Proportions of Severity levels of SCD With Length of Hospital Stay Across the Year of Service (2009-2022)

		Major			Moderate			Minor		
**Year**	**Total**	**n**	**Proportion (%)**	**Mean (SD) Length of Stay**	**n**	**Proportion (%)**	**Mean (SD) Length of Stay**	**n**	**Proportion (%)**	**Mean (SD) Length of Stay**
2,009	5,912	754	12.8	10.2 (11.7)	2,271	38.4	5.7 (4.7)	2,887	48.8	4.1 (3.4)
2,010	6,028	921	15.3	9.4 (9.0)	2,362	39.2	5.6 (4.5)	2,745	45.5	4.0 (3.3)
2,011	2,012	409	20.3	9.1 (11.4)	847	42.1	5.6 (4.9)	756	37.6	4.0 (3.1)
2,012	2,604	574	22.0	8.6 (9.8)	1,081	41.5	5.2 (4.5)	949	36.4	3.9 (3.3)
2,013	2,981	441	14.8	10.0 (12.3)	1,243	41.7	5.8 (5.2)	1,297	43.5	3.8 (3.2)
2,014	3,068	561	18.3	10.0 (11.8)	1,271	41.4	5.5 (4.6)	1,236	40.3	3.9 (3.5)
2,015	2,980	594	19.9	10.0 (13.6)	1,128	37.9	5.6 (6.0)	1,258	42.2	3.7 (3.3)
2,016	2,870	588	20.5	9.0 (11.4)	1,106	38.5	5.0 (4.6)	1,176	41.0	3.5 (2.8)
2,017	4,340	1,004	23.1	7.8 (8.0)	1,639	37.8	5.3 (4.2)	1,697	39.1	3.5 (2.8)
2,020	5,976	1,414	23.7	8.9 (10.0)	2,562	42.9	5.3 (4.8)	2,000	33.5	3.9 (3.4)
2,022	3,771	1,022	27.1	9.7 (12.4)	1,514	40.2	5.4 (5.1)	1,235	32.8	3.9 (3.8)
**Test for equality of proportions**		**$\chi^2_{(10)}$ = 540.4, p-value < 0.001**			**$\chi^2_{(10)}$ = 58.1, p-value < 0.001**			**$\chi^2_{(10)}$ = 494.0, p-value < 0.001**		

2018, 2019, and 2021 were dropped due to missingness of total costs in those years of service in the SPARCS dataset

© 2026 Iloegbu C et al. *JAMA Network Open*.

eTable 3. Marascuilo Comparison of Severity Levels of SCD Across the Health Service Areas (2009-2022)

Comparison	Major			Moderate			Minor		
	Value	Critical range	Significance	Value	Critical range	Significance	Value	Critical range	Significance
Capital/Adirond-Central NY	0.021	0.039	no	0.008	0.053	no	0.013	0.053	no
Capital/Adirond-Finger Lakes	0.035	0.035	no	0.077	0.048	yes	0.042	0.048	no
Capital/Adirond-Hudson Valley	0.005	0.033	no	0.009	0.044	no	0.014	0.044	no
Capital/Adirond-Long Island	0.047	0.048	no	0.042	0.06	no	0.005	0.06	no
Capital/Adirond-New York City	0.044	0.03	yes	0.014	0.039	no	0.031	0.039	no
Capital/Adirond-Southern Tier	0.057	0.126	no	0.056	0.146	no	0.001	0.151	no
Capital/Adirond-Western NY	0.056	0.039	yes	0.082	0.048	yes	0.026	0.049	no
Central NY-Finger Lakes	0.015	0.033	no	0.069	0.047	yes	0.054	0.047	yes
Central NY-Hudson Valley	0.026	0.031	no	0.001	0.042	no	0.026	0.042	no
Central NY-Long Island	0.068	0.047	yes	0.05	0.059	no	0.018	0.059	no
Central NY-New York City	0.065	0.027	yes	0.022	0.038	no	0.043	0.038	yes
Central NY-Southern Tier	0.077	0.125	no	0.064	0.146	no	0.013	0.15	no
Central NY-Western NY	0.077	0.036	yes	0.09	0.047	yes	0.013	0.048	no
Finger Lakes-Hudson Valley	0.04	0.026	yes	0.068	0.037	yes	0.028	0.036	no
Finger Lakes-Long Island	0.082	0.044	yes	0.119	0.055	yes	0.037	0.055	no
Finger Lakes-New York City	0.079	0.021	yes	0.091	0.031	yes	0.011	0.03	no

© 2026 Iloegbu C et al. *JAMA Network Open*.

Comparison	Major			Moderate			Minor		
	Value	Critical range	Significance	Value	Critical range	Significance	Value	Critical range	Significance
Finger Lakes-Southern Tier	0.092	0.124	no	0.133	0.145	no	0.041	0.149	no
Finger Lakes-Western NY	0.092	0.033	yes	0.159	0.042	yes	0.068	0.042	yes
Hudson Valley-Long Island	0.042	0.042	no	0.051	0.051	no	0.009	0.052	no
Hudson Valley-New York City	0.039	0.018	yes	0.023	0.024	no	0.017	0.023	no
Hudson Valley-Southern Tier	0.052	0.123	no	0.065	0.143	no	0.013	0.147	no
Hudson Valley-Western NY	0.051	0.031	yes	0.091	0.036	yes	0.04	0.038	yes
Long Island-New York City	0.003	0.039	no	0.028	0.047	no	0.026	0.048	no
Long Island-Southern Tier	0.01	0.128	no	0.014	0.149	no	0.004	0.153	no
Long Island-Western NY	0.01	0.046	no	0.04	0.054	no	0.031	0.056	no
New York City-Southern Tier	0.012	0.122	no	0.042	0.142	no	0.03	0.146	no
New York City-Western NY	0.012	0.027	no	0.069	0.031	yes	0.056	0.032	yes
Southern Tier-Western NY	0	0.125	no	0.026	0.144	no	0.026	0.149	no

© 2026 Iloegbu C et al. *JAMA Network Open*.

eTable 4. Marascuilo Comparison of Severity Levels of SCD in New York State Across the Year of Service (2009-2022)

Comparison	Major			Moderate			Minor		
	Value	Critical range	Significance	Value	Critical range	Significance	value	Critical range	Significance
2009-2010	0.025	0.020	yes	0.033	0.028	yes	0.008	0.027	no
2009-2011	0.076	0.031	yes	0.113	0.039	yes	0.037	0.039	no
2009-2012	0.093	0.028	yes	0.124	0.035	yes	0.031	0.036	no
2009-2013	0.020	0.024	no	0.053	0.034	yes	0.033	0.034	no
2009-2014	0.055	0.025	yes	0.085	0.034	yes	0.030	0.034	no
2009-2015	0.072	0.026	yes	0.066	0.034	yes	0.006	0.034	no
2009-2016	0.077	0.027	yes	0.079	0.035	yes	0.001	0.034	no
2009-2017	0.104	0.024	yes	0.097	0.030	yes	0.006	0.030	no
2009-2020	0.109	0.022	yes	0.154	0.027	yes	0.045	0.028	yes
2009-2022	0.143	0.026	yes	0.161	0.031	yes	0.017	0.031	no
2010-2011	0.050	0.031	yes	0.080	0.039	yes	0.029	0.039	no
2010-2012	0.068	0.029	yes	0.091	0.035	yes	0.023	0.035	no
2010-2013	0.005	0.025	no	0.020	0.034	no	0.025	0.034	no
2010-2014	0.030	0.026	yes	0.053	0.034	yes	0.022	0.034	no
2010-2015	0.047	0.027	yes	0.033	0.034	no	0.013	0.034	no
2010-2016	0.052	0.027	yes	0.046	0.034	yes	0.006	0.034	no
2010-2017	0.079	0.024	yes	0.064	0.030	yes	0.014	0.030	no
2010-2020	0.084	0.022	yes	0.121	0.027	yes	0.037	0.028	yes
2010-2022	0.118	0.026	yes	0.128	0.031	yes	0.010	0.031	no

© 2026 Iloegbu C et al. *JAMA Network Open*.

Comparison	Major			Moderate			Minor		
	Value	Critical range	Significance	Value	Critical range	Significance	value	Critical range	Significance
2011-2012	0.017	0.037	no	0.011	0.044	no	0.006	0.045	no
2011-2013	0.055	0.034	yes	0.059	0.043	yes	0.004	0.044	no
2011-2014	0.020	0.035	no	0.027	0.043	no	0.007	0.044	no
2011-2015	0.004	0.036	no	0.046	0.043	yes	0.042	0.044	no
2011-2016	0.002	0.036	no	0.034	0.044	no	0.036	0.044	no
2011-2017	0.028	0.034	no	0.015	0.040	no	0.043	0.041	yes
2011-2020	0.033	0.032	yes	0.041	0.038	yes	0.008	0.039	no
2011-2022	0.068	0.036	yes	0.048	0.041	yes	0.019	0.042	no
2012-2013	0.072	0.032	yes	0.071	0.040	yes	0.002	0.041	no
2012-2014	0.038	0.033	yes	0.038	0.040	no	0.001	0.040	no
2012-2015	0.021	0.034	no	0.058	0.040	yes	0.037	0.040	no
2012-2016	0.016	0.034	no	0.045	0.041	yes	0.030	0.041	no
2012-2017	0.011	0.032	no	0.027	0.037	no	0.037	0.037	no
2012-2020	0.016	0.030	no	0.030	0.035	no	0.014	0.036	no
2012-2022	0.051	0.034	yes	0.037	0.037	no	0.014	0.039	no
2013-2014	0.035	0.029	yes	0.032	0.039	no	0.003	0.039	no
2013-2015	0.051	0.030	yes	0.013	0.039	no	0.038	0.039	no
2013-2016	0.057	0.031	yes	0.025	0.040	no	0.032	0.039	no
2013-2017	0.083	0.028	yes	0.044	0.036	yes	0.039	0.036	yes
2013-2020	0.089	0.026	yes	0.100	0.034	yes	0.012	0.034	no
2013-2022	0.123	0.030	yes	0.108	0.037	yes	0.015	0.037	no

© 2026 Iloegbu C et al. *JAMA Network Open*.

Comparison	Major			Moderate			Minor		
	Value	Critical range	Significance	Value	Critical range	Significance	value	Critical range	Significance
2014-2015	0.016	0.031	no	0.019	0.039	no	0.036	0.039	no
2014-2016	0.022	0.032	no	0.007	0.039	no	0.029	0.039	no
2014-2017	0.048	0.029	yes	0.012	0.036	no	0.037	0.036	yes
2014-2020	0.054	0.027	yes	0.068	0.033	yes	0.014	0.034	no
2014-2022	0.088	0.031	yes	0.075	0.036	yes	0.013	0.037	no
2015-2016	0.006	0.032	no	0.012	0.040	no	0.007	0.039	no
2015-2017	0.032	0.030	yes	0.031	0.036	no	0.001	0.036	no
2015-2020	0.037	0.028	yes	0.087	0.034	yes	0.050	0.034	yes
2015-2022	0.072	0.032	yes	0.095	0.036	yes	0.023	0.037	no
2016-2017	0.026	0.030	no	0.019	0.036	no	0.008	0.036	no
2016-2020	0.032	0.029	yes	0.075	0.034	yes	0.043	0.034	yes
2016-2022	0.066	0.032	yes	0.082	0.037	yes	0.016	0.037	no
2017-2020	0.005	0.026	no	0.056	0.030	yes	0.051	0.030	yes
2017-2022	0.040	0.030	yes	0.064	0.033	yes	0.024	0.033	no
2020-2022	0.034	0.028	yes	0.007	0.030	no	0.027	0.032	no

2018, 2019, and 2021 were dropped due to missingness of total costs in those years of service in the SPARCS dataset

© 2026 Iloegbu C et al. *JAMA Network Open*.

eTable 5. Dunn HSD Multiple Comparisons for Length of Stay, Total Charges, and Total Costs Across Health Service Areas

		Length of Stay			Total Charges			Total Costs		
**HSA 1**	**HSA 2**	Estimate	Adj. P	Sig. level	Estimate	Adj. P	Sig. level	Estimate	Adj. P	Sig. level
Capital/Adirond	Central NY	4.409	< 0.001	***	6.220	< 0.001	****	12.919	< 0.001	****
Capital/Adirond	Finger Lakes	0.111	1.000	ns	-16.834	< 0.001	****	-2.302	0.597	ns
Capital/Adirond	Hudson Valley	6.027	< 0.001	****	19.650	< 0.001	****	19.018	< 0.001	****
Capital/Adirond	Long Island	1.496	1.000	ns	23.594	< 0.001	****	16.875	< 0.001	****
Capital/Adirond	New York City	0.907	1.000	ns	17.266	< 0.001	****	23.611	< 0.001	****
Capital/Adirond	Southern Tier	-2.413	0.443	ns	-5.424	< 0.001	****	-1.662	1.000	ns
Capital/Adirond	Western NY	-0.590	1.000	ns	-9.558	< 0.001	****	5.804	< 0.001	****
Central NY	Finger Lakes	-4.859	< 0.001	****	-24.354	< 0.001	****	-16.942	< 0.001	****
Central NY	Hudson Valley	0.771	1.000	ns	12.637	< 0.001	****	3.663	0.007	**
Central NY	Long Island	-2.299	0.602	ns	18.622	< 0.001	****	5.978	< 0.001	****
Central NY	New York City	-5.251	< 0.001	****	9.318	< 0.001	****	6.536	< 0.001	****
Central NY	Southern Tier	-3.971	0.002	**	-7.627	< 0.001	****	-6.211	< 0.001	****
Central NY	Western NY	-5.495	< 0.001	****	-16.731	< 0.001	****	-8.351	< 0.001	****
Finger Lakes	Hudson Valley	7.096	< 0.001	****	45.742	< 0.001	****	25.875	< 0.001	****
Finger Lakes	Long Island	1.534	1.000	ns	39.911	< 0.001	****	20.307	< 0.001	****
Finger Lakes	New York City	0.984	1.000	ns	48.416	< 0.001	****	33.747	< 0.001	****
Finger Lakes	Southern Tier	-2.481	0.367	ns	-0.033	1.000	ns	-0.937	1.000	ns
Finger Lakes	Western NY	-0.806	1.000	ns	8.080	< 0.001	****	9.306	< 0.001	****
Hudson Valley	Long Island	-3.229	0.035	*	11.157	< 0.001	****	3.895	0.003	**
Hudson Valley	New York City	-9.688	< 0.001	****	-7.897	< 0.001	****	3.779	0.004	**
Hudson Valley	Southern Tier	-4.270	0.001	***	-11.423	< 0.001	****	-7.388	< 0.001	****
Hudson Valley	Western NY	-7.810	< 0.001	****	-35.385	< 0.001	****	-14.680	< 0.001	****
Long Island	New York City	-1.150	1.000	ns	-15.797	< 0.001	****	-2.391	0.471	ns
Long Island	Southern Tier	-2.969	0.084	ns	-14.840	< 0.001	****	-8.441	< 0.001	****
Long Island	Western NY	-2.110	0.977	ns	-33.494	< 0.001	****	-13.214	< 0.001	****
New York City	Southern Tier	-2.734	0.175	ns	-10.245	< 0.001	****	-8.072	< 0.001	****
New York City	Western NY	-2.016	1.000	ns	-35.838	< 0.001	****	-20.100	< 0.001	****
Southern Tier	Western NY	2.247	0.690	ns	2.340	0.540	ns	3.593	0.009	**
**ns = not significant, * = significant at 0.05, ** = significant at 0.01, *** = significant at 0.005, **** = significant at 0.001**										

© 2026 Iloegbu C et al. *JAMA Network Open*.
