# Supplementary material for: Geographic and Temporal Differences in Sickle Cell Disease Hospitalizations in New York State
Source: JAMA Netw Open. 2026 May 1;9(5):e2610045. doi: 10.1001/jamanetworkopen.2026.10045 (PMC13135208; doi:10.1001/jamanetworkopen.2026.10045)
Supplement: Supplement 2. — Data Sharing Statement [file jamanetwopen-e2610045-s002.pdf]

Data Sharing Statement

Iloegbu. Geographic and Temporal Differences in Sickle Cell Disease Hospitalizations in New York State. *JAMA Netw Open*. Published May 01, 2026.
doi:10.1001/jamanetworkopen.2026.10045

Data
Data available: Yes
Data types: Deidentified participant data, Data dictionary, Other (please specify)
Additional Information: Publicly available de-identified administrative data from the New York State Department of Health's Statewide Planning and Research Cooperative System (SPARCS).
How to access data: https://www.health.ny.gov/statistics/sparcs/
When available: With publication

Supporting Documents
Document types: None

Additional Information
Who can access the data: The data are publicly available to any qualified researcher or member of the public through the New York State Department of Health's Statewide Planning and Research Cooperative System (SPARCS) Open Data portal.
Types of analyses: The dataset may be used for epidemiological, health services, and outcomes research, including analyses of hospitalization trends, healthcare utilization, and regional or demographic disparities in health outcomes.
Mechanisms of data availability: Signed data access agreement
